# Noncontact Monitoring and Imaging of the Operation and Performance of Thin‐Film Field‐Effect Transistors

**DOI:** 10.1002/advs.202407923

**Published:** 2024-11-21

**Authors:** Kwangsik Jeong, Dong yeob Shin, Ji‐Min Park, Dong‐Joon Yi, Hyunmin Hong, Hyun‐Suk Kim, Kwun‐Bum Chung

**Affiliations:** ^1^ Division of AI Semiconductor Yonsei University Wonju 26493 Republic of Korea; ^2^ Department of Physics Dongguk University Seoul 04620 Republic of Korea; ^3^ Department of Energy and Materials Engineering Dongguk University Seoul 04620 Republic of Korea

**Keywords:** amorphous oxide semiconductor, methodology & inspection, second harmonic generation

## Abstract

In this study, the first noncontact and non‐destructive methodology is developed for monitoring and imaging the operation and performance of thin‐film field‐effect transistors (TFTs) using second‐harmonic generation (SHG) imaging. By analyzing the SHG signal intensity, which is directly related to the electric field at the interface between the semiconductor channel and gate insulator, critical electrical parameters such as the threshold voltage (V_TH_) and flat‐band voltage (V_FB_) are successfully determined. These findings demonstrate a strong correlation between SHG signals and V_TH_ and V_FB_ in InGaZnO TFTs under various process conditions. Notably, the method achieves an unprecedented resolution of ΔV_FB_ below 100 mV in assessing electrical properties through SHG measurements, surpassing conventional spectroscopy techniques. Furthermore, a system is developed to monitor and image the TFT array operation and performance, enabling us to distinguish between pass and fail devices and measure the V_TH_ distribution based on the SHG intensity. This approach facilitates early failure detection and supports efficient curing during manufacturing, thereby marking significant advancements in TFT technology and quality control processes.

## Graphical abstract

This study examines the electrical properties of AOS‐TFTs using SHG signals to measure V_TH_ and V_FB_. SHG measurements assess the electrical properties of AOS‐TFTs with high sensitivity. A system is developed for non‐contact, non‐destructive monitoring of TFT performance using SHG imaging, enabling early failure detection and improved manufacturing processes.

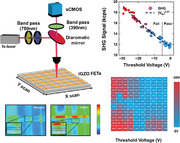

## 1 Introduction

Next‐generation immersive displays, such as holograms and augmented/virtual reality displays, have revolutionized the display industry. These advanced displays require smaller pixels and higher pixel density (PPI) because of their close proximity to viewers.^[^ ^1^, ^2^ ^]^ Simultaneously, panel sizes are increasing, with Generation 10.5 panels reaching dimensions of 2.94 m × 3.37 m.^[^ ^3^ ^]^ As the industry evolves in both directions, the number of thin‐film transistors (TFTs) required has increased. To ensure uniform turn‐on voltage and pixel brightness, the electrical performance must be consistent across this vast number of TFTs. However, achieving this in amorphous oxide semiconductors (AOSs) is challenging because of variations in composition, oxygen deficiency, annealing temperature, and impurities.^[^ ^4^ ^]^

Current compensation circuits address brightness discrepancies in individual circuits; however, the size of these circuits restricts the achievable PPI.^[^ ^5^ ^]^ Consequently, methods for monitoring or investigating the electrical properties of TFTs during the backend process are becoming increasingly important. Traditional electrical measurements and spectroscopic analyses in the backplane struggle with the scale and sensitivity required for large, dense arrays.^[^ ^6^ ^]^

Furthermore, optical analysis of pixel brightness can only be performed after the entire panel is processed, complicating the application of post‐processing techniques, such as UV or laser annealing, to correct manufacturing defects.^[^ ^6^, ^7^ ^]^ Therefore, a new, noncontact, and highly sensitive method is necessary to evaluate the electrical properties of these devices.

In this study, we introduce a second‐harmonic generation (SHG) method for measuring the electrical properties of AOS TFTs with high sensitivity.^[^ ^8^ ^]^ SHG detects subtle changes in key parameters, such as the flat‐band voltage, making it highly effective for identifying minor electrical variations across devices. We developed a prototype system for noncontact analysis of TFT arrays, providing large‐scale, high‐resolution mapping of electrical properties, which is essential for the future of high‐PPI and large‐area displays.

## 2 Results and Discussion

**Figure 1:**
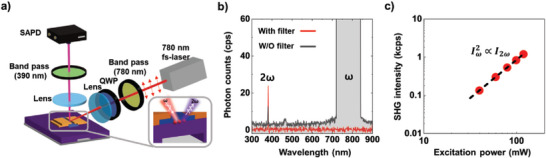
a) Diagram of second‐harmonic generation (SHG) measurement system for single thin‐film field‐effect transistor (TFT) device. Circularly polarized femtosecond laser with 780 nm wavelength is focused on an InGaZnO (IGZO) channel at 45° angle, and the obtained SHG signal is measured using a single‐photon detector after blocking reflected rays of 780 nm using a bandpass filter. b) Spectrum of optical signal with and without bandpass filters. By using a bandpass filter, only SHG signals are measured in the optical spectrometer. c) Incidence power‐dependent intensity of the SHG signal. SHG signal is proportional to square of the incident laser intensity.

**Figure** **1**a) illustrates the experimental setup for measuring the SHG in an AOS TFT. The setup includes a circularly polarized femtosecond laser (fs‐laser) that is positioned at an angle of 45° to the substrate and generates an incident ray with a wavelength of 780 nm at pulse durations of 150 fs, which is aimed at the channel area of the AOS TFT. The resulting 390 nm SHG signal is measured at the same angle using a single‐photon counter. To isolate the SHG signal, two bandpass filters with an optical density (OD) of six block the reflected or scattered light at 780 nm. Figure 1b) illustrates the differences in the intensity of wavelength‐dependent light measured using a spectrometer with and without bandpass filters. The filters could effectively block light with frequency ω (780 nm wavelength), reducing its intensity below detection limits. Figure 1c) shows the relationship between the power of the incident light and SHG intensity. The results confirm that the SHG intensity is proportional to the square of the fundamental power, showing a quadratic relationship between the incident light and SHG intensities ($I_{\omega }^{2}\propto I_{2\omega }$).^[^ ^9^ ^]^

**Figure 2:**
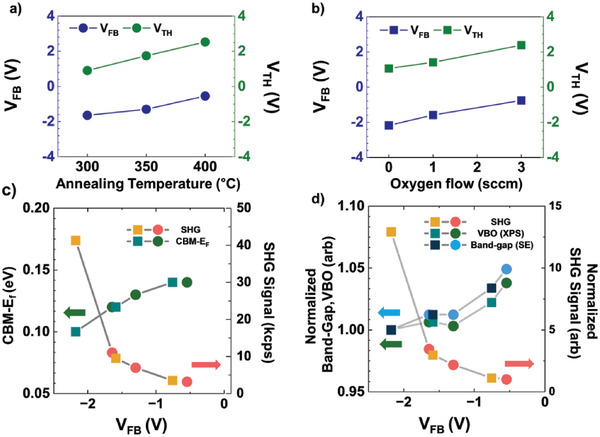
Variations in electrical properties of TFTs with IGZO active channel with changes in (a) annealing temperature and b) oxygen flow. As the partial pressure of oxygen during deposition or post‐annealing temperature increases, flat‐band voltage (V_FB_) and threshold voltage (V_TH_) are increased. c) (left axis) V_FB_‐dependent difference between conduction band maximum and Fermi level (CBM−Ef). CBM−E_f_ represents carrier concentration in IGZO and depends on V_FB_. (right axis) V_FB_ ‐dependent intensity of SHG. d) (left axis) Normalized SHG intensity, bandgap (from spectroscopic ellipsometry), valence band offset (from X‐ray photoelectron spectroscopic image of valence band) versus V_FB_ of devices and (right axis) normalized V_FB_‐dependent intensity of SHG. Circular symbols represent samples for which the annealing temperature was controlled, whereas square symbols represent samples for which the oxygen flow was controlled.

To determine the relationship between the electronic properties of AOS TFTs and their SHG signals, we prepared AOS TFTs with an InGaZnO (IGZO) active channel layer. **Figure** **2**a,b) show the changes in key transfer parameters, such as the threshold and flat‐band voltages, in response to changes in the annealing temperature and oxygen flow rate, respectively. These IGZO layers were prepared under varying conditions, including different annealing temperatures and oxygen flow rates, to obtain various electrical properties (the transfer characteristics of the IGZO TFTs are shown in Figure S1, Supporting Information). Notably, the oxygen flow during deposition and annealing temperature in ambient air significantly influence the defect states in oxide semiconductors. In particular, increasing the annealing temperature or partial pressure of oxygen reduces oxygen‐related defects in IGZO thin films. Because oxygen vacancies (VOs) are electron donors within these semiconductors, decreasing VO results in a positive shift in both the threshold and flat‐band voltages.^[^ ^4a,b^ ^]^

To validate the consistency using traditional methods and compare sensitivity differences, we conducted spectroscopic analyses and SHG signal measurements on thin films and AOS TFTs under identical processing conditions. Figure 2c) shows the V_FB_‐dependent difference between the conduction band maximum and Fermi level (CBM−E_F_) on the left axis and V_FB_‐dependent SHG intensity on the right axis. The CBM−E_F_ can be evaluated from the valence band maximum (VBM) in the valence band spectra obtained using X‐ray photoelectron spectroscopy (XPS) and the bandgap obtained using spectroscopic ellipsometry (SE). This difference indicates the carrier density and is directly related to the V_TH_ or V_FB_ of the active channel materials. Thus, the electrical properties of IGZO can be approximately estimated using the CBM−E_F_.^[^ ^10^ ^]^ The CBM − E_F_ increases from 0.1 to 0.14 eV when V_FB_ increases from −2.18 to −0.55 V. Simultaneously, the intensity of the SHG varies from 3.5 to 40 kilocounts per second (kcps) when the V_FB_ varies from −0.55 to −2.18 V. Therefore, both spectroscopic method and measurement of SHG intensity can be used to evaluate the electrical properties of AOS TFTs. However, CBM − E_F_ has a positive correlation with V_FB_, whereas SHG intensity exhibits a negative correlation with V_FB_.

Figure 2d shows the normalized SHG intensity, bandgap (obtained using SE), and valence band offset (from the valence band spectra obtained using XPS) corresponding to the V_FB_ of the devices. Through XPS and SE, CBM−E_F_, which is related to both V_FB_ and V_TH_, can be evaluated. Whereas XPS and SE measure the energy‐level differences that affect the Fermi level and electron count, SHG measures the interfacial electric field, with the SHG signal increasing as V_FB_ shifts further from zero.The change in CBM−E_F_ between devices is related to the energy resolution limits of spectroscopy; consequently, distinguishing the differences in electrical properties of devices in which ΔV_TH_ or ΔV_FB_ < 1 V is difficult. When V_FB_ varies from −2.18 to −0.55 V, the measured VBM and bandgap only varied by 5%, whereas a 10‐fold variation in the intensity of SHG is observed. Therefore, the electrical properties of AOS TFTs can be more precisely estimated using SHG intensity. Furthermore, spectroscopy typically has a measurement area diameter greater than 100 µm, which makes it impractical to obtain electrical properties from individual devices. Because measurements require tens of seconds to several minutes, spectroscopy is better suited for analyzing the physical properties of thin films than for examining the electrical characteristics of individual devices.

To investigate the relationship between the SHG intensity and electrical properties of the AOS, we analyzed the electrical properties (flat‐band and threshold voltages) and SHG intensity.

When *V_FB_* < 0 < *V_TH_*, the intensity of the SHG signal at *V_g_* = 0 is as follows (Material S1, Supporting Information):^[^ ^11^ ^]^

(1) $$I_{2\omega }\propto {\left(-V_{FB}\right)}^{4\left(\frac{T_{deep}}{T}-1\right)},\;\mathrm{for}\;V_{FB}<0<V_{TH}\;at\;V_{g}=0$$

Moreover, when *V_TH_* < 0,  the intensity of SHG signal at *V_g_* = 0 is as follows

(2) $$I_{2\omega }\propto {\left(V_{TH}\right)}^{4\left(\frac{T_{tail}}{T}-1\right)},\;\mathrm{for}\;V_{TH}<0\;\mathrm{at}\;\;V_{G}=0$$

where I_2ω_, *T, T_tail_*, and *T_deep_* are the intensity of SHG, temperature, and characteristic temperatures in deep and tail states, respectively.

**Figure 3:**
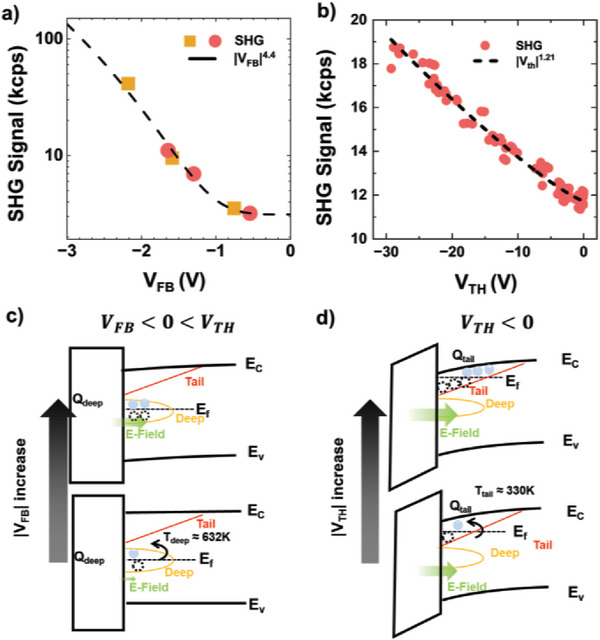
a) Relation between V_FB_ and SHG intensity in *V_FB_* < 0 < *V_TH_* region. In this case, SHG intensities are proportional to |*V_FB_* |^4.4^. Circular symbols represent the samples for which the annealing temperature was controlled, whereas square symbols represent the samples for which the oxygen flow was controlled. b) Relation between V_TH_ and SHG intensity in *V_TH_* < 0 region. In this case, SHG intensities are proportional to |*V_TH_* |^1.21^. c) Diagram showing interface electric field for *V_FB_* < 0 < *V_TH_* region. In this region, carrier is activated in deep states in the amorphous oxide semiconductor (AOS). d) Diagram showing interface electric field for *V_TH_* < 0 region. In this region, carrier is activated in tail states in the AOS.

**Figure** **3**a) shows the V_FB_‐dependent SHG intensity of devices under various process conditions. As all devices satisfied the condition *V_FB_* < 0 < *V_TH_*, the intensity of SHG signals should be described using Equation (1). Therefore, we plotted the relation between V_FB_ and SHG intensity for devices under various process conditions. The relation between V_FB_ and SHG intensity can be modeled based on |*V_FB_* |^4.4^ at T_deep_ = 630 K, which comparable to the previously reported value for the characteristic temperature in deep states.^[^ ^11c^ ^]^

In this region, carriers are generated by activation in deep‐level states, and the difference between the gate bias and V_FB_ modulates the carrier density, as shown in Figure 3c). Note that when the annealing temperature increased from 300 to 400 °C or oxygen flow during deposition increased from 0 to 3 sccm, the SHG signal intensity from the device reduced. This reduction in the SHG intensity corresponds to a decrease in the carrier density in deep‐level states, which is related to the density of oxygen defects. Higher annealing temperatures or increased oxygen flows diminish the density of oxygen vacancies, thereby decreasing the carrier density within the AOS. Consequently, the Fermi level in the AOS shifts downward, and flat‐band voltage approaches 0 V, indicating a decrease in the interface electric field. The relation between the SHG intensity and  *V_FB_* of devices under various annealing temperatures and oxygen flows can be fitted into a single equation, and the intensity of SHG exhibits a 10‐fold variation when  *V_FB_* is changed from −1.5 to −0.5 V. Therefore, the electrical properties of AOS can be estimated from the SHG intensity.

Figure 3b) shows the V_TH_‐dependent SHG intensity of the device array with random irradiation with femtosecond‐UV laser irradiation to obtain devices with various electrical properties (wide range of V_TH_) and *V_TH_* < 0. The SHG intensity was obtained using an imaging and scanning system, which is introduced in the subsequent section. As shown in Figure 3b), the relation between V_TH_ and the SHG intensity can be modeled based on |*V_TH_* |^1.21^ at T_tail_ = 390 K, which corresponds to a previously reported value of the characteristic temperature of tail states.^[^ ^11c^ ^]^ In this case, the generated carrier density in the tail state is high, and the difference between gate bias and *V_TH_* primarily affects the electric field at the interface. In this region, charges are primarily generated by carrier activation in tail states, as shown in Figure 3d). Therefore, the characteristic temperature corresponds to the previously reported characteristic temperatures for tail states within IGZO.

In both scenarios (V_FB_ < 0 < V_TH_ or V_TH_ < 0), the electrical properties of the AOS TFTs and their SHG intensities are linked to carrier activation in the sub‐bandgap states (either tail or deep states) within the AOS. However, the increase factor follows the characteristic temperature of the states within the bandgap where the carriers are activated. As the interface electric field in AOS TFTs is affected by the process conditions and/or post‐processing adjustments, changes in the SHG intensity effectively reflect these modifications. Consequently, SHG intensity measurement provides a noncontact and nondestructive method for evaluating the electrical properties of AOS TFTs, enabling precise adjustments and diagnostics without damaging the devices.

**Figure 4:**
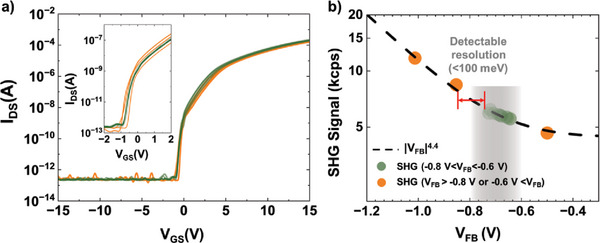
a) Transfer curves of devices fabricated under the same conditions. The flat‐band voltages for most devices are within the range of −0.8 V < V_FB_ < −0.6 V. The inset shows the magnified transfer curves of devices. b) V_FB_ versus SHG intensity of devices fabricated under the same process conditions. Variation of Δ V_FB_ ≈ 0.1 V can be accurately identified via SHG measurement.

Currently, IGZO‐based TFTs exhibit a V_TH_ close to zero (V_FB_ around a few 100 mV in the negative) and a V_TH_ shift of several 100 mV due to electrical stress or defects.^[^ ^12^ ^]^ To verify whether it is possible to distinguish such small differences, we measured the electrical properties and SHG intensity for a device array processed under identical conditions. **Figure** **4**a) shows the transfer curves of a device array processed under identical conditions, and Figure 4b) shows the relationship between V_FB_ and SHG intensity for each device fabricated under identical process conditions. Because the high resolution of SHG measurements enables the precise estimation of electrical properties, the differentiation of devices with marginal variations is possible. As shown by the transfer curves in Figure 4a), most devices have a V_FB_ ranging from −0.8 to −0.6 V; however, certain devices exhibit V_FB_ values greater than −0.6 V or less than −0.8 V. The SHG intensity trends exhibited by these devices are identical, as shown in Figure 4b). Within the V_FB_ range of −0.8 to −0.6 V, the SHG intensities are recorded between 5.5 and 6.2 kcps. For V_FB_ values of −1.0, −0.85, and −0.5 V, the SHG intensities are 12.2, 8.4, and 0.45 kcps, respectively. Moreover, the V_FB_ and SHG intensities are proportional to |V_FB_|^4.4^, as discussed previously. This demonstrates that SHG measurements can accurately detect variations in the V_FB_ of ≈0.1 V. This capability allows SHG measurements to serve as a diagnostic tool for identifying differences in the electrical properties of devices fabricated in the same batch.

**Figure 5:**
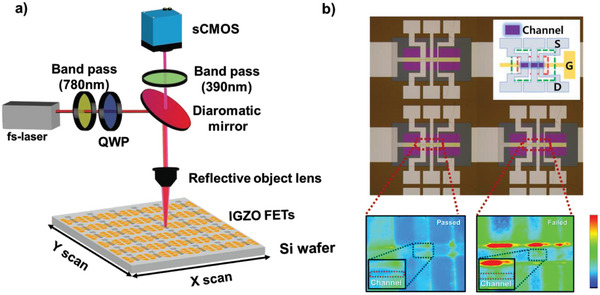
a) Diagram of a scanning system for SHG signals of AOS TFT arrays. A femtosecond laser with 780 nm wavelength is focused using a reflective objective lens, and the reflected beam is blocked with a dichroic mirror and bandpass filters. Images of SHG are obtained using a sensitive scientific complementary metal‐oxide semiconductor camera. b) Optical image of AOS TFTs and SHG images for passed and failed devices. The SHG intensity of active channels in TFTs is intensive for failed devices with large negative V_TH_.

In modern display panels, differences of several volts in V_TH_ within the pixel circuit can be controlled by a compensation circuit.^[^ ^5^, ^7^, ^13^ ^]^ Therefore, to inspect for defects in the TFTs of a display panel, it is crucial to quickly detect devices with V_TH_ differences exceeding several volts. To measure the circuit in one go and rapidly identify devices with significant V_TH_ differences, we developed a system that measures the SHG signal using an sCMOS camera. **Figure** **5**a) illustrates the imaging and scanning system used to measure the SHG signals for an array of AOS TFTs. An fs‐laser is focused on each devices using a reflective objective lens; moreover, a dichroic mirror and bandpass filters are used to effectively eliminate the scattering or reflection of 780 nm (ω) light. A scientific complementary metal‐oxide semiconductor camera captures the SHG signals from each TFT. Device selection is facilitated using an XY translation stage. Figure 5b) shows an optical image of the AOS TFT array and the SHG intensity images obtained using this imaging and scanning system. Our device contains three TFTs in a single cell. The TFTs in a single cell have various channel widths (1, 3, and 5 µm) and a channel length of 3 µm. The electrical properties of the TFTs were obtained from devices with 3 µm channel width. As shown in the figure, the SHG intensity in the channel region of the passed device is uniform and low, whereas the SHG intensity of the failed device is non‐uniform and higher. The SHG intensity was notably stronger in devices with larger negative V_TH_ values than in those with smaller negative V_TH_ values (the transfer curves for several devices are shown in Figure S6, Supporting Information)

The theoretical limitation of spatial resolution based on Abbe's law for an SHG signal (390 nm) with an NA of 0.5 is 400 nm. Therefore, estimating electrical properties using SHG imaging is suitable for TFTs in display panels, where the channel length of FETs is generally over 1 µm. Using this system, a 10 × 10 devices array was scanned to establish the relationship between V_TH_ and the average SHG intensity of the channel area, demonstrating the utility of this method for assessing the variations in electrical properties across multiple devices.

**Figure 6:**
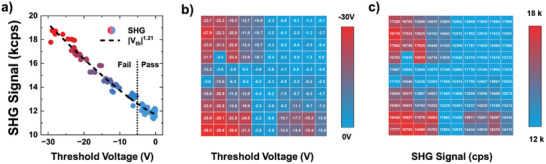
a) Relation between V_TH_ and SHG intensity for 10 × 10 array of TFTs. b) Map of V_TH_ for 10 × 10 array of TFTs. c) Map of SHG intensity for 10 × 10 array of TFTs. The distribution of electrical properties of TFTs (or the position of failed devices) can be evaluated with a distribution of SHG intensities.

**Figure** **6**a) illustrates the results for V_TH_ as measured from the transfer curves of each TFT, along with the SHG signals obtained from the average intensity in the channel area (which is consistent with the data shown in Figure 3b)). In this figure, the devices are categorized as “pass” or “fail” based on their V_TH_ values. Devices with |V_TH_| < 5 V were considered to have passed, whereas those with |V_TH_| > 5 V were considered to have failed considering the performance of compensation circuit. According to the figure, the passed devices with |V_TH_| < 5 V exhibit SHG intensities between 10 and 12 kcps, whereas the failed devices with |V_TH_| > 5 V show SHG intensities ranging from 12 to 19 kcps. For the condition where V_TH_ < 0 and V_FB_ < 0, the SHG signal is proportional to |V_TH_|^1.21^ within the range of 0 V < V_TH_ < −30 V. This correlation enables the mapping of the V_TH_ and SHG intensities across the wafer. By measuring the V_TH_ and SHG intensities at each position on the wafer, a map was created to visualize the failed devices distribution and electrical properties. Figure 6b,c) show the V_TH_ and SHG intensity maps for the device array, respectively. These maps reveal that devices with higher SHG intensities tend to have larger |V_TH_| values, and the position of the failed devices, as well as the |V_TH_| distribution within the array, can be inferred from the SHG signal distribution. Using SHG signals induced by an fs‐laser, the electronic properties of devices on a wafer scale can be effectively determined and visualized, enabling noncontact, nondestructive evaluation of device performance and quality.

## 3 Conclusion

In this study, we developed a novel noncontact and nondestructive methodology for monitoring and imaging the operation and performance of oxide TFTs using the relationship between device properties and SHG signals. The threshold and flat‐band voltages, related to the density of oxygen vacancies and active carriers, are significantly important parameters of AOS TFTs for device operation and performance. V_TH_ and V_FB_ were analyzed by measuring the signal and intensity of the SHG affected by the electric field at the interface between the semiconductor channel and gate insulator. Consequently, the SHG measurement determines the device operation (pass/fail) as a function of V_TH_ or V_FB_, and we could demonstrate the distinction between devices when Δ V_FB_ was below 100 mV. Based on two representative densities of states (tail and deep states) in AOS active channel, the SHG intensity was quantitatively analyzed using two characteristic equations as a function of temperature and V_TH_ (*V_TH_* < <0) or V_FB_ (*V_FB_* < 0 < *V_TH_*) with a power of $4(\frac{T_{tail}}{T}-1)$ or $4(\frac{T_{deep}}{T}-1)$ under various processing conditions, such as post‐annealing temperature, oxygen partial pressure, and UV irradiation. In addition, defect‐related stress‐induced shifts in V_TH_ or V_FB_ can be measured by monitoring changes in SHG intensity under optical stress, as shown in Materials S9 and S8 (Supporting Information). These results demonstrate the possibility of measuring deep‐level defect‐related phenomena such as subthreshold swing (SS), device stability, mobility, and on‐currents

Moreover, a system for monitoring and imaging the electrical operation and performance of a TFT array was constructed by scanning the SHG intensity. Using this system, the failed devices were clearly distinguished from the passed devices, and the distribution of the device performance across a large area was obtained. Using the method proposed in this study, the device properties of a TFT and its array can be evaluated without physical contact or destruction. Consequently, device failures can be detected during the fabrication process. Although our experiments were conducted using a bottom‐gate structure, the methods we propose can also be applied to a top‐gate structure. As shown in the process flows in Figure S9 (Supporting Information), an AOS/GI structure is present, along with an inspection process utilizing optical methods at an earlier stage. As demonstrated in Material S11 (Supporting Information), the SHG intensity of the AOS/GI‐only structure also reflects the electronic properties of the AOS/GI/Metal structure. Another option is optical access from the substrate, as shown in Figure S10 (Supporting Information). When a glass substrate with SiN or SiO_2_ buffer layers is used, the fs‐laser (780 nm) and SHG signal (390 nm) can be transmitted through the backside. Therefore, major TFT structures in display panels, such as Bottom‐Gate, Top‐Contact (BG‐TC), Bottom‐Gate, Bottom‐Contact (BG‐BC), and Top‐Gate, Bottom‐Contact (TG‐BC), can be measured.

Electrical measurements using a probe card and multiple instruments have speed limitations when inspecting the characteristics of all TFTs on large panels. These limitations become more pronounced as the panel size increase and the TFT density increase, such as with a 300 PPI display containing 20000 TFTs, where inspection using 100 electrical instruments would take ≈200 s, excluding the contact time. In contrast, optical measurements using a high‐power laser and CCD‐based SHG imaging can significantly reduce inspection time.

In addition, electrical measurements require the fabrication of metal contact pads, meaning that electrical property inspections can only occur after source and drain electrode patterning, which involves multiple process steps following the fabrication of the AOS/GI structure. The process costs can be significantly reduced by performing SHG measurements at an earlier stage. Furthermore, whereas conventional contact‐based IV measurements for selected TEGs are limited to investigating only a few devices, SHG enables noncontact, non‐destructive inspection of all FETs on the panel, offering a more comprehensive evaluation.

## 4 Experimental Section

### Device Fabrication

Transistor devices were fabricated on a Si wafer using thermally grown SiOx. The thickness of the oxidized layer was 300 nm. First, a sputtering system deposited 150 nm thick Al as the gate electrode. Next, a 300 nm thick SiO_2_ film was formed as the gate dielectric via plasma‐enhanced chemical vapor deposition. A dry etching process created the gate electrode and gate dielectric patterns. Subsequently, 30 nm thick IGZO active layers were deposited at room temperature via sputtering with an IGZO target (In:Ga:Zn = 1:1:1 at%). The working pressure was 2 mTorr, plasma power was 400 W. Moreover, the Ar gas flow rate was fixed at 50 sccm, while the O gas flow rate was varied from 0 to 3 sccm. Finally, 150 nm thick source and drain Al electrodes were deposited via sputtering. An i‐line UV source (365 nm) with a stepper process was used for photolithography to form small patterns. When patterning the active layer and source/drain electrode, a lift‐off process was used to prevent damage to the underlying layer. Thermal annealing was conducted from 300 to 400 °C for 1 h in air ambient.

### Second‐Harmonic Generation (SHG)

To measure the SHG of a single device, a 780 nm (ω) fs‐laser with 120 mW power,150 fs pulse width, and 80 MHz repetition rate was irradiated to the channel area of the IGZO TFT. A linearly polarized fs‐laser was converted to a circularly polarized laser using a λ/4 waveplate. Using a circularly polarized laser, the effect of the Si crystal structure was flattened. Using two bandpass filters with a center wavelength (CWL) of 780 nm and OD of six, the SHG from the laser was blocked. A plano‐convex lens with a focal length of 75 mm was used to focus the 780 nm laser on the channel and collect the SHG signal. A 780 nm fs‐laser was positioned at 45° to the substrate before irradiation, and the 390 nm (2ω) signal was measured at an angle of 45° to the substrate. To block reflected or scattered 780 nm (ω) light, two bandpass filters with 390 nm CWL and OD of six were used. The intensity of the SHG was measured using a Si Avalanche photodetector with 15% photon detection efficiency at 400 nm wavelength. The SHG intensity was measured and averaged for 100 s.

### SHG Imaging

An SHG microscope that can control the incident angle and an XY translation stage were constructed to image the electrical properties of AOS devices using SHG. Similar to the single‐device measurement, a 780 nm (ω) fs‐laser with 120 mW power,150 fs pulse width, and 80 MHz repetition rate was irradiated. Using two bandpass filters with 780 nm CWL and OD of six, the SHG signals from the laser were blocked before the laser passed through an optical fiber provided with the fs‐laser. The 780 nm fs‐laser was focused using a reflective‐type objective at 40x magnification. The reflected or scattered 780 nm (ω) light was blocked using a dichroic mirror and bandpass filters with 390 nm CWL and OD of six. To determine the V_TH_ from the SHG image, only the SHG signal magnitude in the channel region of the device was averaged.

## Conflict of Interest

The authors declare no conflict of interest.

## Supporting information



Supporting Information

## Data Availability

The data that support the findings of this study are available from the corresponding author upon reasonable request.
