# Supplementary material for: Noncontact Monitoring and Imaging of the Operation and Performance of Thin‐Film Field‐Effect Transistors
Source: Adv Sci (Weinh). 2024 Nov 21;12(5):2407923. doi: 10.1002/advs.202407923 (PMC11791970; doi:10.1002/advs.202407923)
Supplement: Supplementary file 1 — Supporting Information [file ADVS-12-2407923-s001.docx]

**Noncontact monitoring and imaging of the operation and performance of thin-film field-effect transistors**

Kwangsik Jeong^1^, Dong-yeop Shin^2^, Ji-Min Park^3^, Dong-Joon Yi^2^, Hyunmin Hong ^1^,
Hyun-Suk Kim^3*^, Kwun-Bum Chung ^2*^

*^1^Division of AI Semiconductor, Yonsei University, Wonju, 26493, Republic of Korea*

*^2^Division of Physics and Semiconductor Science Dongguk University, Seoul 04620, Republic of Korea*

*^3^Department of Energy and Materials Engineering, Dongguk University, Seoul 04620, Republic of Korea*

**Supporting material 1.**

At the interface between two materials (semiconductor and gate insulator in a field-effect transistor (FET)), the intensity of SHG is related to the electric field (E-field) at the interface, which is given by the following equation :

$I_{2\omega}= \left| \chi_{2}+\chi_{3}\left[ E_{\mathrm{dc}} \right] \right|^{2} I_{\omega}^{2},$

where $I_{2\omega}$ and $I_{2\omega}$ denote the intensities of the SHG and fundamental light. $\chi_{2}$ and $\chi_{3}$ denote the second- and third-order susceptibilities.$E_{\mathrm{DC}}$ is the E-field intensity at the interface.

The E-field at an interface can be obtained from the relationship between the interface charge density and interface E-field using the Gaussian law. The interface E-field at the surface can be obtained using the following equation:

Q=$\frac{E_{DC}}{\varepsilon_{GI}}.$

Therefore, the E-field at the interface, which is related to the intensity of the SHG signal, can be obtained from the charge at the surface. At the interface of the gate insulator and AOS, the charges at the interface can be simplified into two components as follows:

$$Q=Q_{tail}+Q_{deep},$$

where $Q_{tail}$ and $Q_{deep}$ denote the charges in the tail and deep states, respectively.

As shown in Supporting Figs. 5a) and b), IGZO has several states in the bandgap. Normally, tail states, which are continuous states near the conduction band edge, and deep states, which are separated from the tail state, primarily affect the electrical properties of a FET with IGZO active channels.

When devices are not turned on ($V_{G}<V_{TH}$) and $V_{FB}<V_{G}$

$Q_{deep}" Q_{tail}$for $V_{FB}<V_{G}<V_{TH}.$

Therefore, the E-field at the interface is primarily affected by $Q_{deep}$ and the charge at the interface is related to the flat-band voltage (V_FB_).

In an AOS semiconductor, carriers are generated by excitation from defect states.

The number of excited carriers can be described based on the characteristic temperature, which is related to carrier activation.

$Q_{deep}\propto{(-V_{FB}+V_{G})}^{\alpha+1}$ for $V_{FB}<V_{G}<V_{TH},$

where $\alpha$ is $2(\frac{T_{deep}}{T}-1)$. Here, $T_{deep}$ is the characteristic temperature of deep states and $T$ is the temperature (room temperature). Because no external bias was applied during the SHG measurement, $V_{G}=0$ and the SHG intensity can be described as follows:

$I_{2\omega}\propto\left( -V_{FB} \right)^{4(\frac{T_{deep}}{T}-1)}$, for $V_{FB}<0<V_{TH}$ at $V_{G}$=0.

As T_deep_ = 632 K for the IGZO,

$I_{2\omega}\propto\left| V_{FB} \right|^{4.4}$ for $V_{FB}<0<V_{TH}$ at $V_{G}$=0.

When the devices are turned on ($V_{TH}<V_{G})$, the charge predominantly exists in the tail states because of the higher density of the tail state than that of the deep state.

$Q_{tail}\gg Q_{deep}$ for $V_{TH}<V_{G}$

Therefore, the E-field at the interface is mainly affected by $Q_{deep}$ and charge at the interface is related to the threshold voltage (V_TH_) as follows:

$Q_{tail}\propto{(-V_{TH}+V_{G})}^{\beta+1}$ for $V_{TH}<0,$

where $\beta$=$(\frac{T_{tail}}{T}-1)$. $T_{tail}$ is the characteristic temperature in the tail state, and $T$ is the temperature (room temperature). The number of excited carriers in the tail state can be described by the characteristic temperature in the tail state, which is related to carrier activation.

Because no external bias was applied during the SHG measurement, $V_{G}=0$ and the SHG intensity can be described as follows:

Q=$E_{DC}/\varepsilon_{GI}$

and

$I_{2\omega}= \left| \chi_{2}+\chi_{3}\left[ E_{\mathrm{dc}} \right] \right|^{2} I_{\omega}^{2}$.

Therefore,

$I_{2\omega}\propto\left( V_{TH} \right)^{4(\frac{T_{tail}}{T}-1)}$, for $V_{TH}<0$ at $V_{G}$=0.

As T_tail_ = 330 K for the IGZO,

$I_{2\omega}\propto\left| V_{FB} \right|^{1.21}$ for $V_{TH}<0$ at $V_{G}$=0.

**Supporting material 2.**


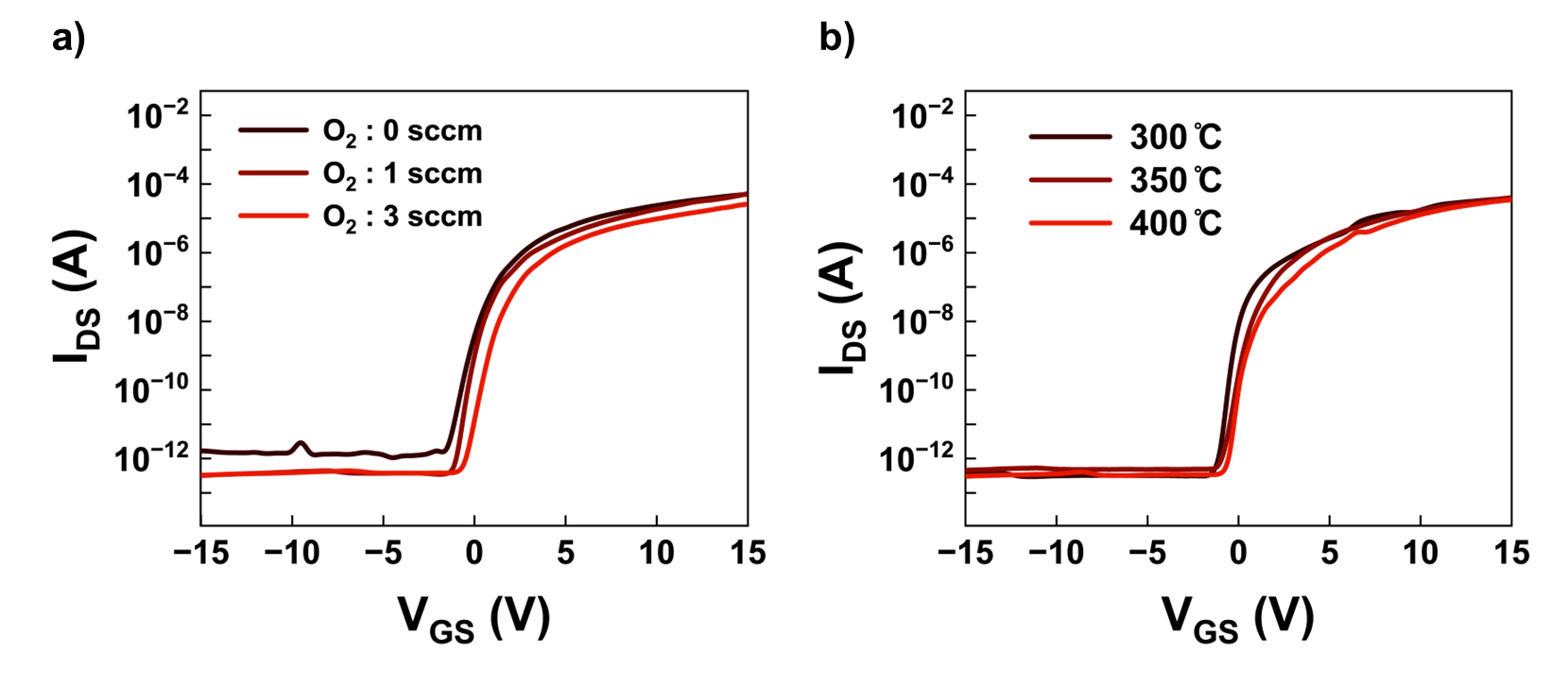


**Supporting Fig. S1　a)** Transfer curves of IGZO field-effect transistors (FETs) under varying oxygen flows during deposition. An increase in the partial pressure of oxygen during deposition results in higher flat-band voltage (V_FB_) and threshold voltage (V_TH_).　**b)** Transfer curves of IGZO FETs under different annealing temperatures. Increase in post-annealing temperatures increases both V_FB_ and V_TH_.

To determine the relationship between electrical properties and SHG in the prepared TFTs with various electrical properties, we fabricated TFTs with an In_1_Ga_1_Zn_1_O_4_ (IGZO) active channel layer. The IGZO channel was synthesized under various conditions, including annealing temperatures and oxygen flow rates. The transfer characteristics of the IGZO TFTs, which are influenced by the annealing temperature and oxygen flow rate, are shown in Supporting Figs. S1 a) and b), respectively. Annealing in ambient air affects the defect states of oxide semiconductors. When the oxygen flow rate was increased during the deposition of the active layer, the density of oxygen-related defects decreased significantly, thereby altering the electrical properties of the IGZO TFTs. As the annealing temperature increased in air, the number of oxygen-related defects in the IGZO thin films decreased. Because VOs act as electron carriers in oxide semiconductors, a reduction in VO results in a positive shift in both the threshold and flat-band voltages.

**Supporting material 3.**

**Supporting Table ST1.** Relative composition of In_1_Ga_1_Zn_1_O_4_ channels


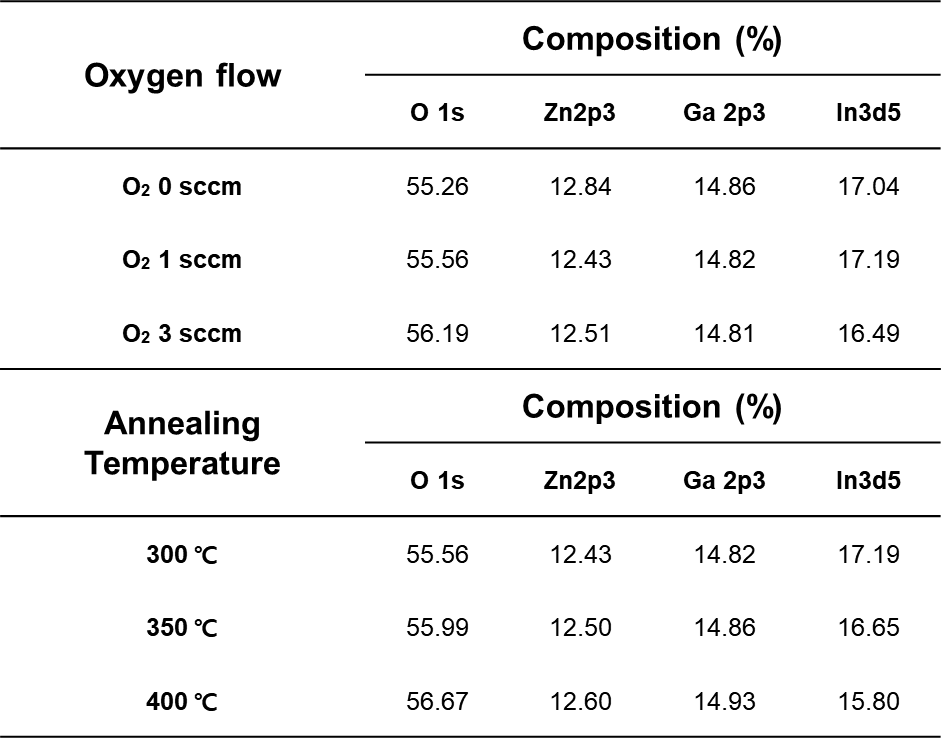


The relative compositions of the IGZO channels under various process conditions are listed in Supporting Table ST1. This composition is evaluated based on the relative intensity of spectra from each core level in XPS spectra. As the oxygen flow rate during deposition increases from 0 to 3 sccm, the oxygen composition increases from 55.26 to 56.19%. An increase in the oxygen flow rate during deposition significantly reduces the density of oxygen-related defects and increases the relative oxygen composition. Similarly, as the post-annealing temperature increases from 300 to 500 °C, the oxygen composition increases from 55.56 to 56.67%. Higher annealing temperatures in the post-annealing process in air cures more oxygen-related defects. Because VOs act as electron carriers in oxide semiconductors, a reduction in VO results in a positive shift in both V_TH_ and V_FB_.

**Supporting material 4.**


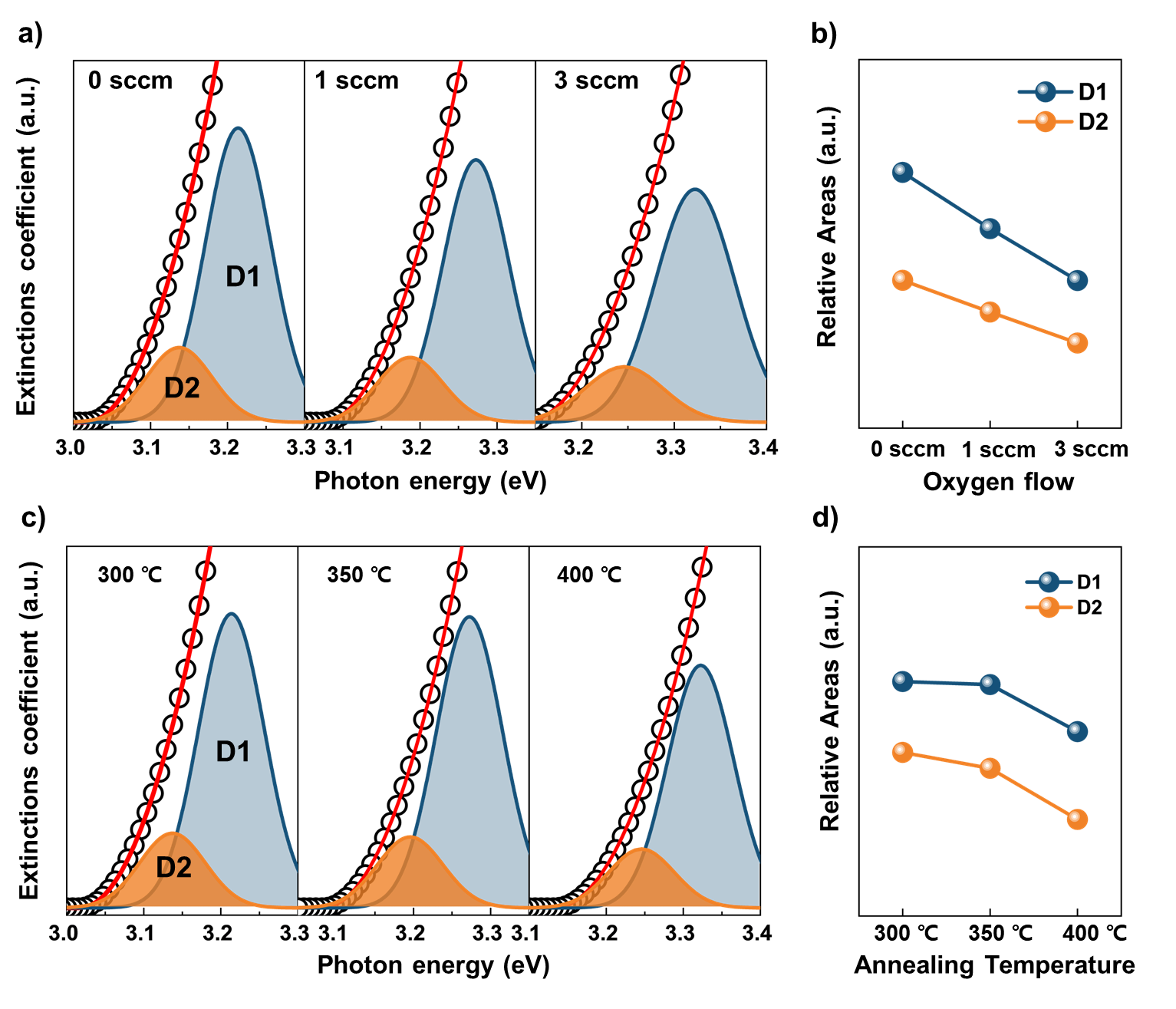


**Supporting Fig. S2 a)** Spectroscopic ellipsometry (SE) spectra of the conduction band for IGZO channels under various oxygen flow rates during deposition. **b)** Relationship between oxygen flow during deposition and the relative area of defect states in the SE spectrum, which is associated with the oxygen vacancy defect density in IGZO. **c)** SE spectra of the conduction band for IGZO channels with different annealing temperatures. **d)** Relationship between the post-annealing temperature and relative area of defect states in SE.

Supporting Figure S2 shows the SE profiles of IGZO under various process conditions and relative areas of the defect states. The pre-edge states can be de-convoluted into D1 and D2: When the oxygen flow during the deposition increases, the density of oxygen-related defects decreases. Consequently, the extinction coefficients in the pre-edge states, representing the oxygen vacancy defects, also decrease. As illustrated in Supporting Fig. S2b), the amounts of both D1 and D2 decrease. Similarly, when the annealing temperature increases, the number of pre-edge states also decreases owing to the curing of oxygen-related defects, as shown in Supporting Figs. S2c) and d).

**Supporting material 5.**


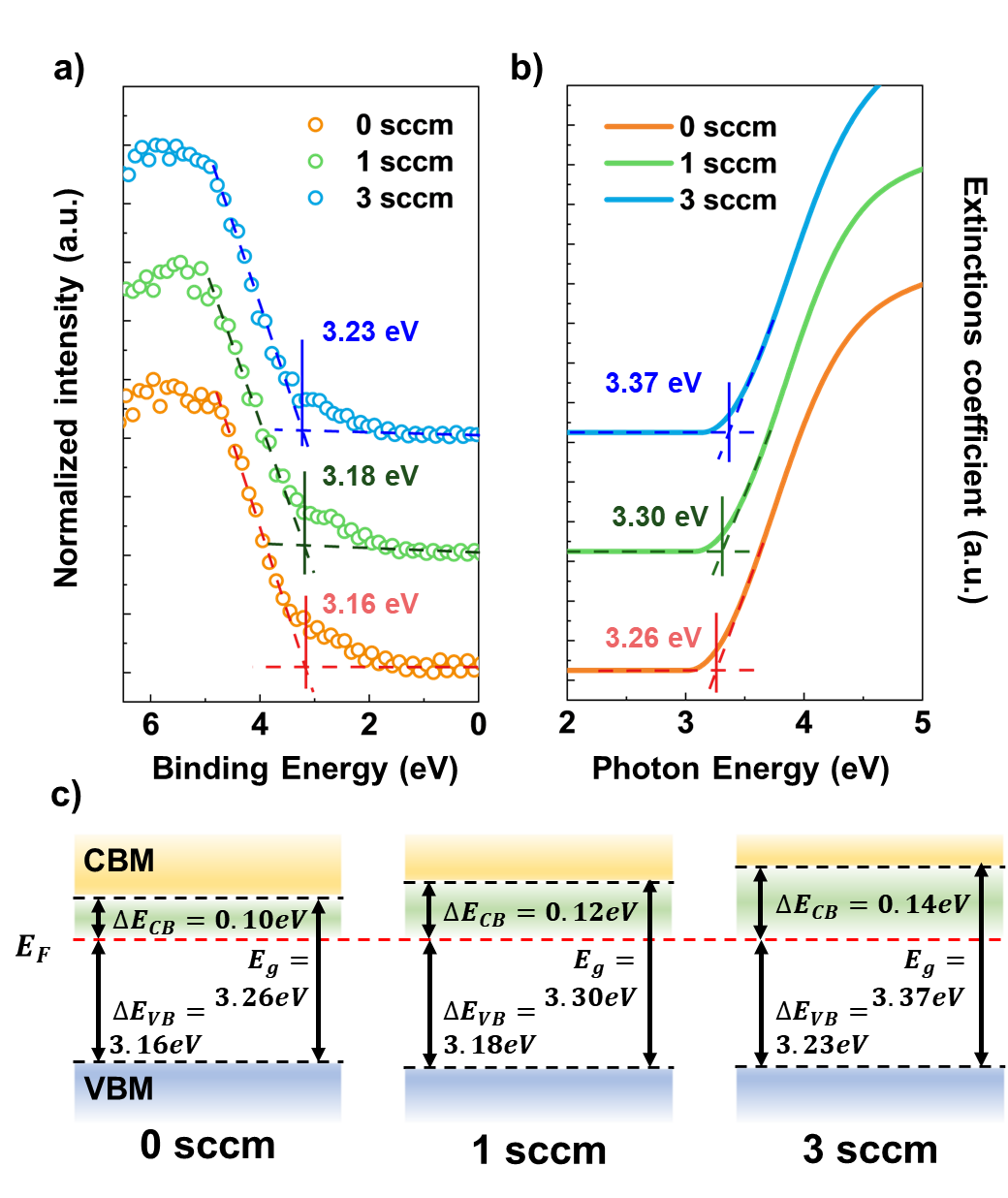


**Supporting Fig. S3 a)** Valence band spectrum from XPS, showing the valence band offset. **b)** Conduction band spectrum obtained using SE, indicating the conduction band minimum or bandgap. **c)** Band alignment derived from both the valence band maximum (VBM) and Fermi level (E_F_) of IGZO samples with varying oxygen flow rates during deposition. As the oxygen flow increases, the $\mathrm{CBM}$ −$E_{F}$, which represents the carrier density of the semiconductor, also increases. A larger CBM − E_F_ corresponds to a lower carrier density in the semiconductor.

Supporting Figure S3 shows the band alignments of IGZOs in which the oxygen flow rates during deposition are controlled. The band alignments were evaluated from the VBO, obtained from the XPS spectra, and the conduction band offset from the SE spectra. CBM − E_F_ can be determined using the maximum difference between the CBM and valence band. This energy difference represents the carrier density in semiconductors. A larger CBM − E_F_ indicates a lower carrier density, whereas a smaller CBM − E_F_ indicates a higher carrier density. As the oxygen flow during deposition increases, CBM − E_F_ increases from 0.10 to 0.14 eV as the oxygen flow increases from 0 to 3 sccm (Supporting Figure 3c). Concurrently, the relative oxygen concentrations increase (refer to Supporting Table ST1), reducing the concentration of VOs in IGZO and suppressing the pre-edge states observed in the SE (illustrated in Supporting Figs. S2 a) and b)).


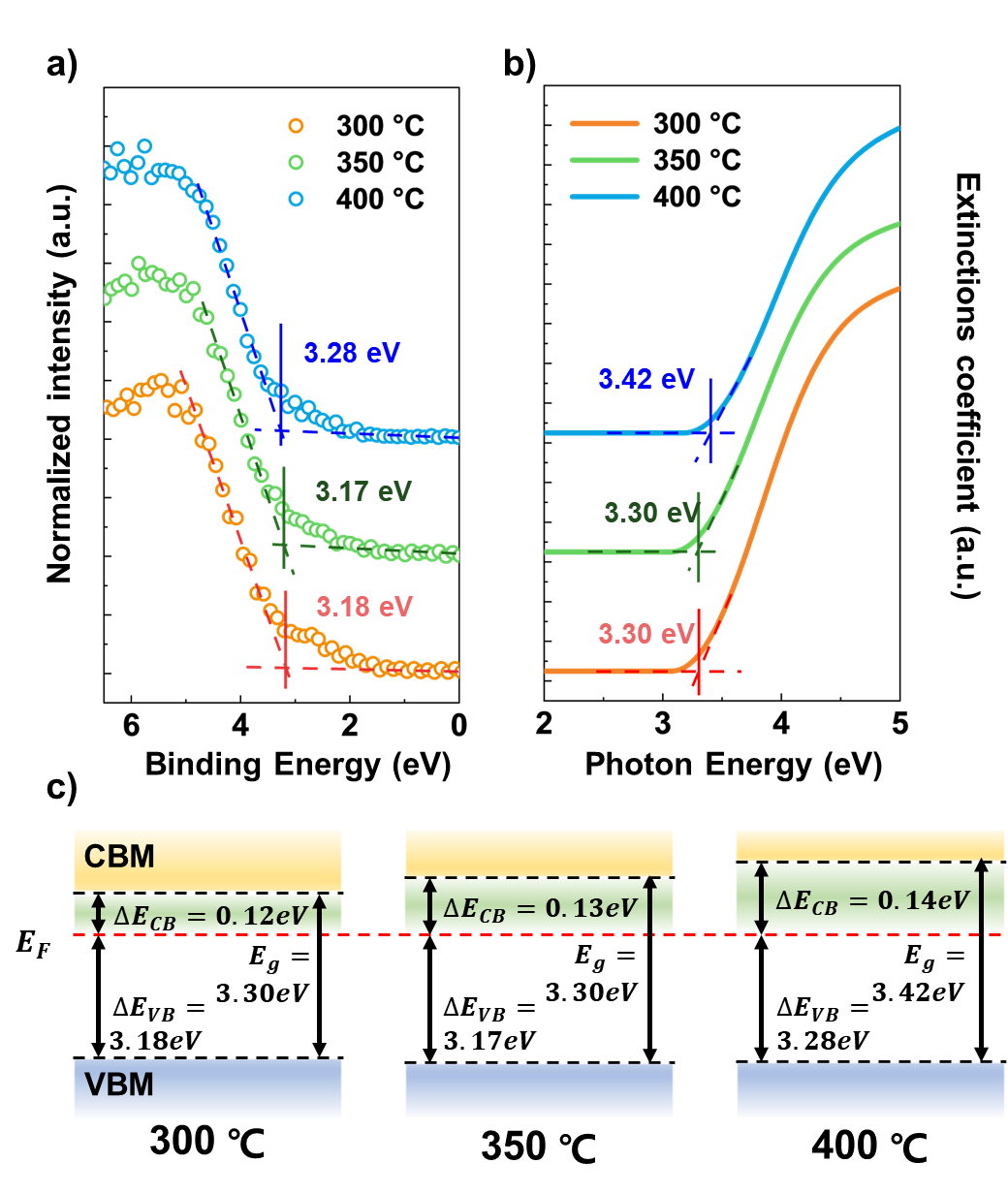


**Supporting Fig. S4 a)** Valence band spectrum obtained from XPS, showing the valence band offset or Fermi level (E_F_). **b)** Conduction band spectrum from SE, indicating the conduction band minimum **c)** Band alignment derived from both the VBM and E_F_ of IGZO samples with varying post-annealing temperatures. As the annealing temperature increases, CBM − E_F_, which represents the carrier density of the semiconductor, also increases.

Supporting Figure S4 shows the band alignments of the IGZOs in which the annealing temperature during the post-annealing process was controlled. As the annealing temperature increases from 300 to 400 ℃, CBM − E_F_ increases from 0.12 to 0.14 eV. Concurrently, the relative oxygen concentrations increase (refer to Supporting Table ST1), reducing the concentration of VOs in IGZO and suppressing the pre-edge states observed in the SE spectra (illustrated in Supporting Figs. S2c) and d)).

**Supporting material 6.**

**
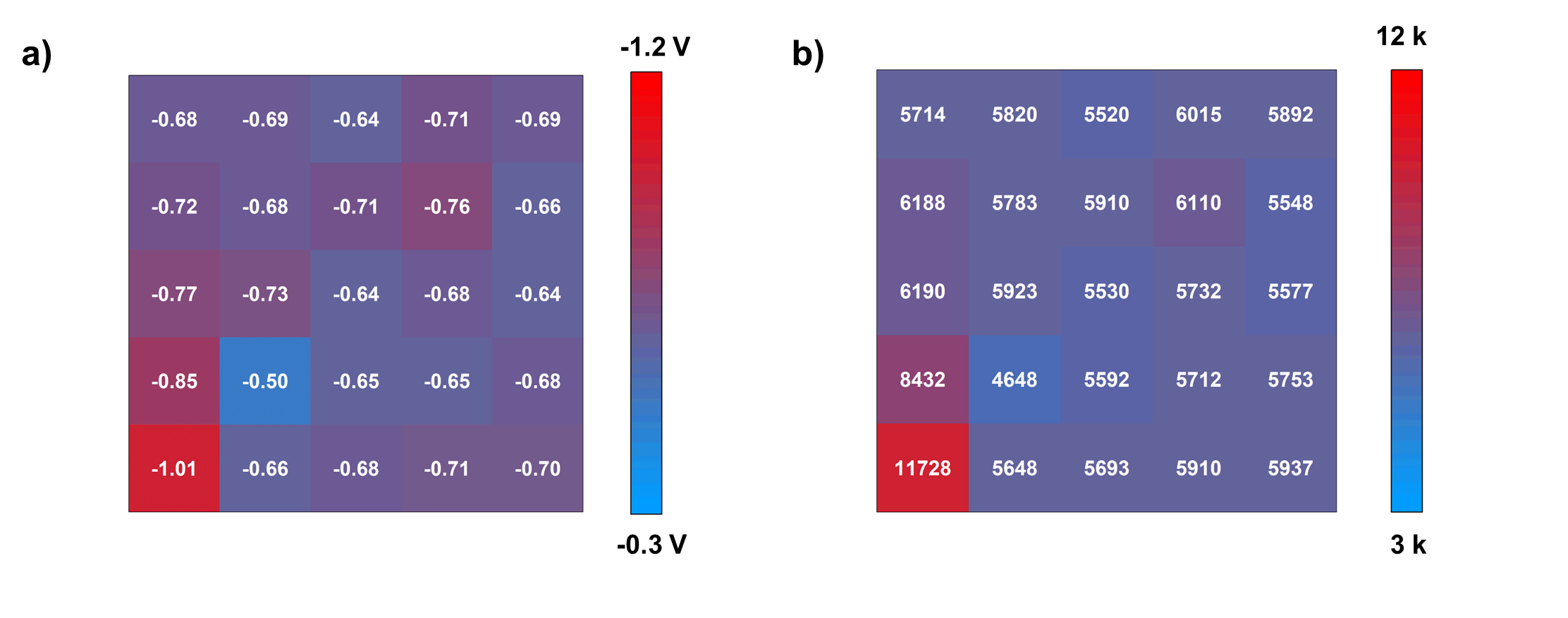
**

**Supporting Fig. S5.** a) Map of V_FB_ for 5 × 5 array of TFTs. b) Map of SHG intensity for 5 × 5 array of TFTs.

Supporting Fig. S5. shows a map of V_FB_ and SHG intensity for a 5 × 5 array on the same wafer. Although the electrical properties among the devices are uniform, the sensitivity of SHG allows the identification of devices where V_FB_ is slightly different from the others

**Supporting material 7.**


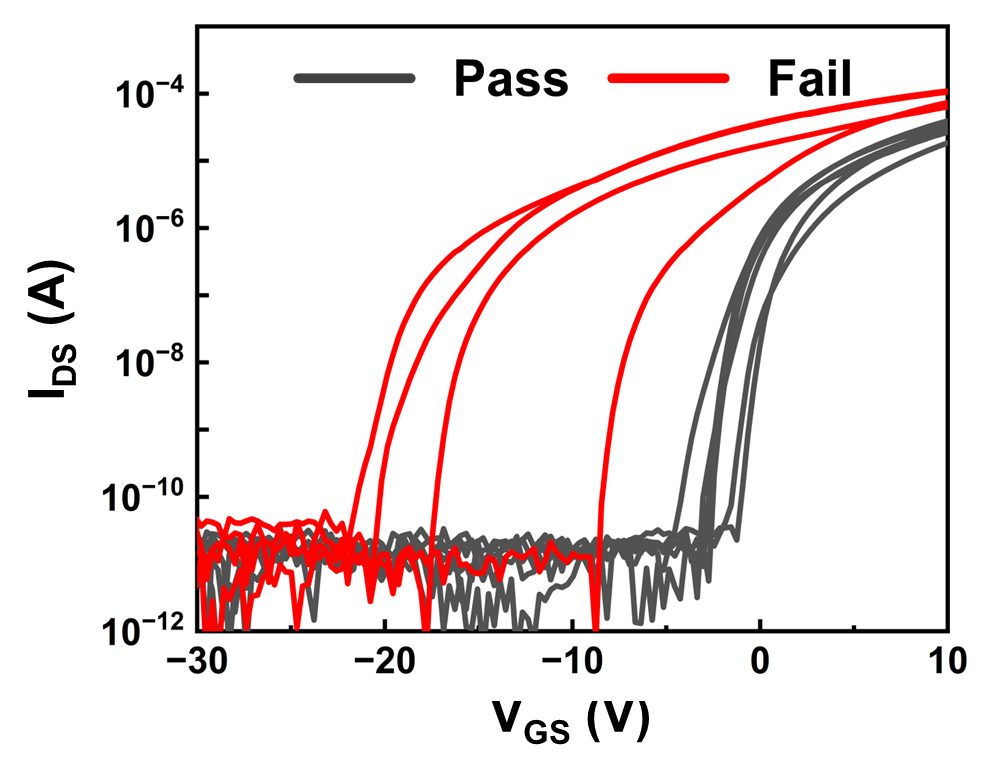


**Supporting Fig. S6:** Transfer curves of failed and passed TFTs, referenced in Fig. 5 of the main manuscript. The failed device exhibits |V_TH_| > 5 V after the post-annealing process with an fs-laser at a 250 nm wavelength, whereas the passed device exhibits a |V_TH_| < 5V.

Supporting Fig. S6 shows the transfer curves of the failed and passed devices. The failed devices were selected from those subjected to fs-UV laser irradiation. The VTH values vary widely due to random irradiation of the fs-UV laser, ranging from −30 to 0 V. The high energy (5 eV) and power (~ 2 W) of the fs-laser generated VOs in the TFTs. Consequently, failed devices, which are always on or exhibit varying brightness, can be simulated. As shown in the figure, some failed devices have V_TH_ values less than –10 V, causing significant differences in the images obtained from SHG signals. Therefore, the failed devices can be distinguished using SHG measurements.

**Supporting material 8.**

**
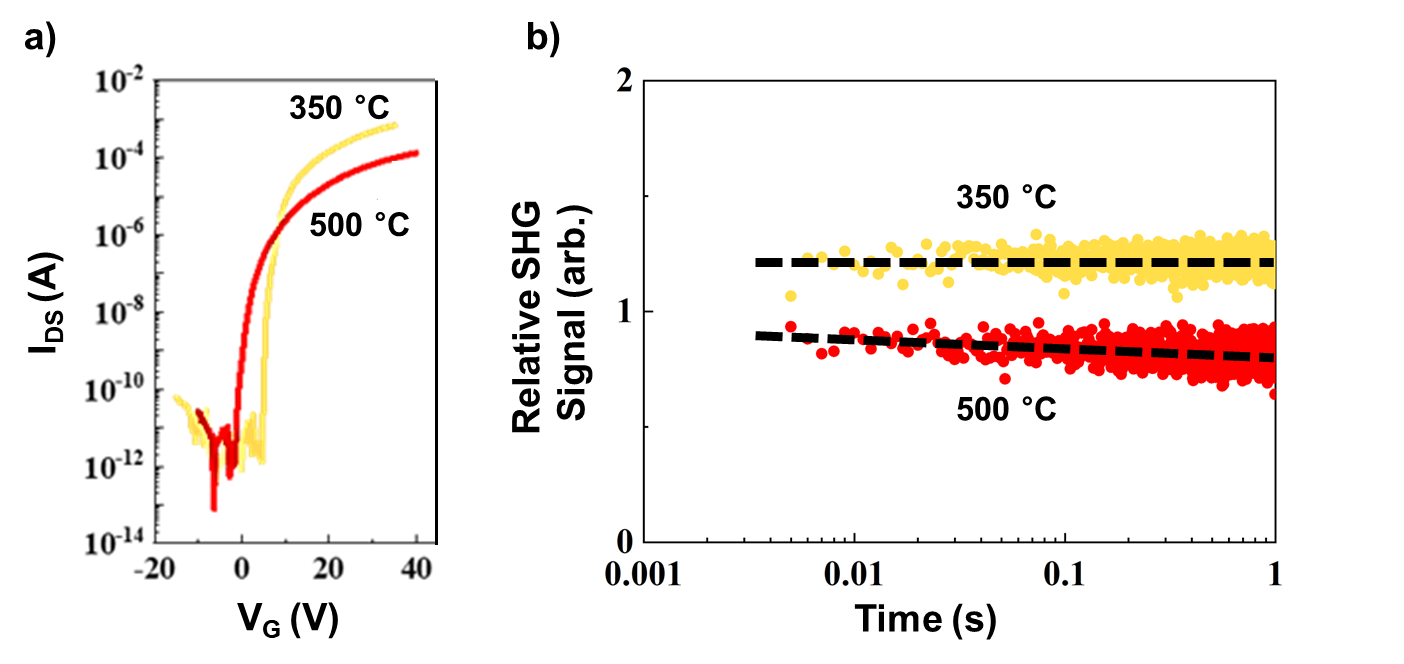
**

**Supporting materials Fig. S7**. a) transfer curve of IGZO device that anneal in 350°C and 500°C in Air. b) time dependent SHG signal within 1 s after laser irradiation.

Because the subthreshold swing (SS) is influenced by the interface trap/de-trap process, differences in SS can be explored through a laser-induced trapping mechanism. Laser irradiation can induce trapping at the interface, leading to changes in the electric field (E-field) intensity at the interface. As the density of defect states increased, the amount of trapped charge also increased, resulting in a more pronounced modulation of the SHG intensity. To confirm this, we measured the changes in the SHG intensity with varying irradiation times, as shown in Supporting Fig. S7. By adjusting the annealing temperature, we fabricated a normal device (annealed at 350 °C) and a degraded device with low mobility and high SS (annealed at 500 °C). The device with low mobility and high SS exhibited a more significant change in SHG intensity owing to charge trapping at the interface.

**Supporting material 9.**


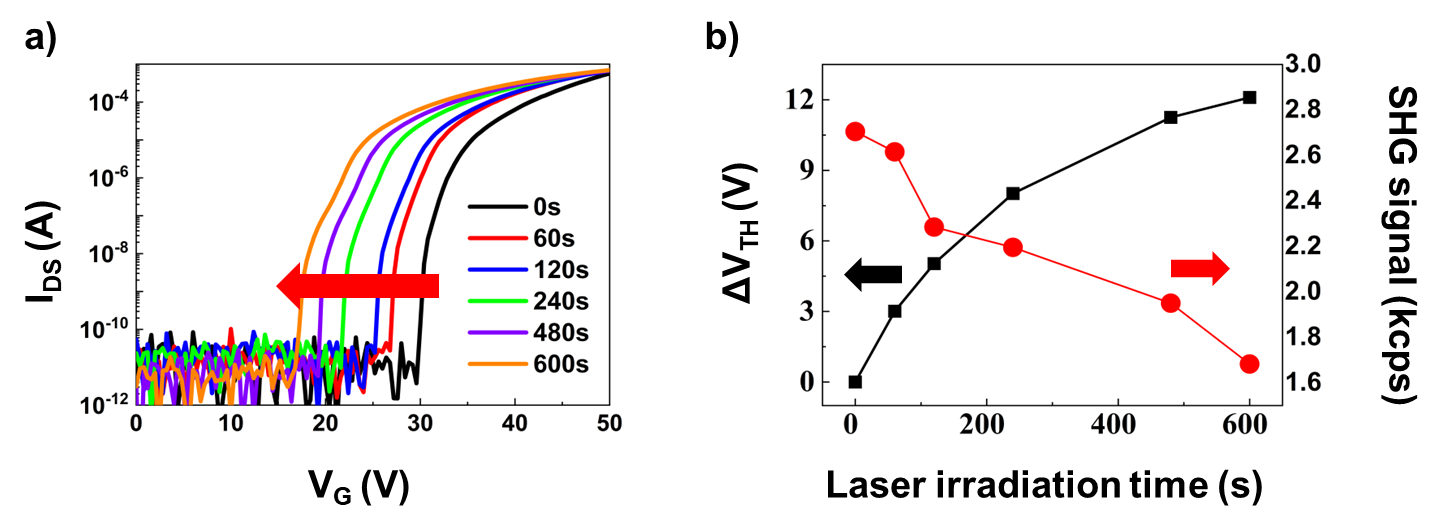


**Supporting materials Fig. S8.** a) transfer curve of IGZO device with laser irradiation.
b) laser irradiation time dependent V_TH_ shift and SHG signal.

Defects in AOS-TFTs cause shifts in V_TH_ because they trap charges under electrical stress, with larger quantities of defects leading to greater V_TH_ shifts. These V_TH_ changes caused by defects are also key evaluation metrics. Similar to electrical stress, optical stress can induce changes in V_TH_. Optical stress can be applied by focusing a strong femtosecond (fs) laser on the device, causing measurable changes in V_TH_. As shown in Supporting Fig. S8, the longer the exposure to the intense laser, the more pronounced the V_TH_ shift. Simultaneously, the SHG signal changes. Therefore, the quantity of defects related to device stability can be detected using the SHG signal induced by strong fs-laser irradiation.

**Supporting material 10.**

**
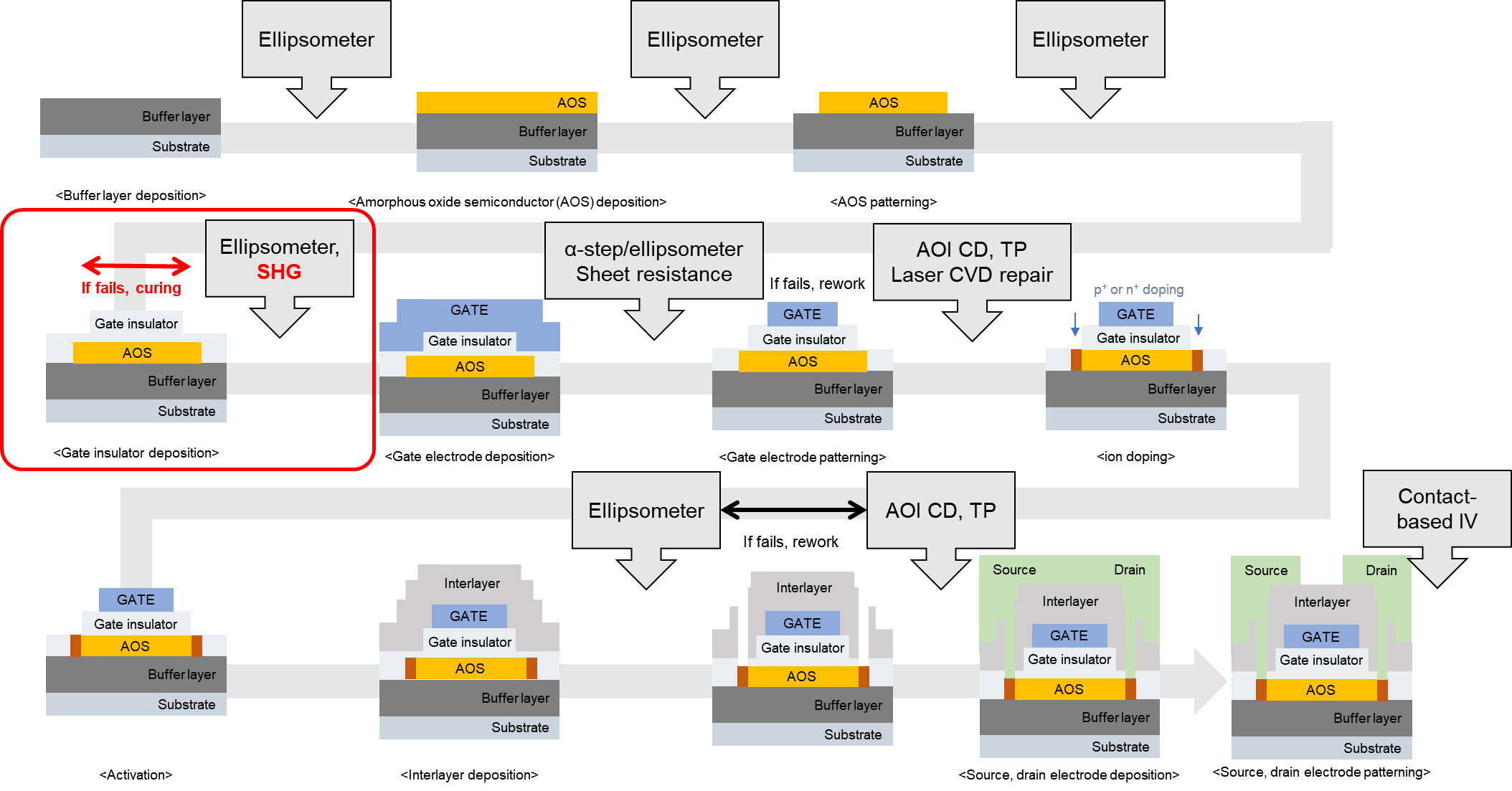
**

**Supporting Fig. S9.** Process flow of TFT in OLED pixels. Our methods can be applied after gate insulator deposition, providing another opportunity for curing.


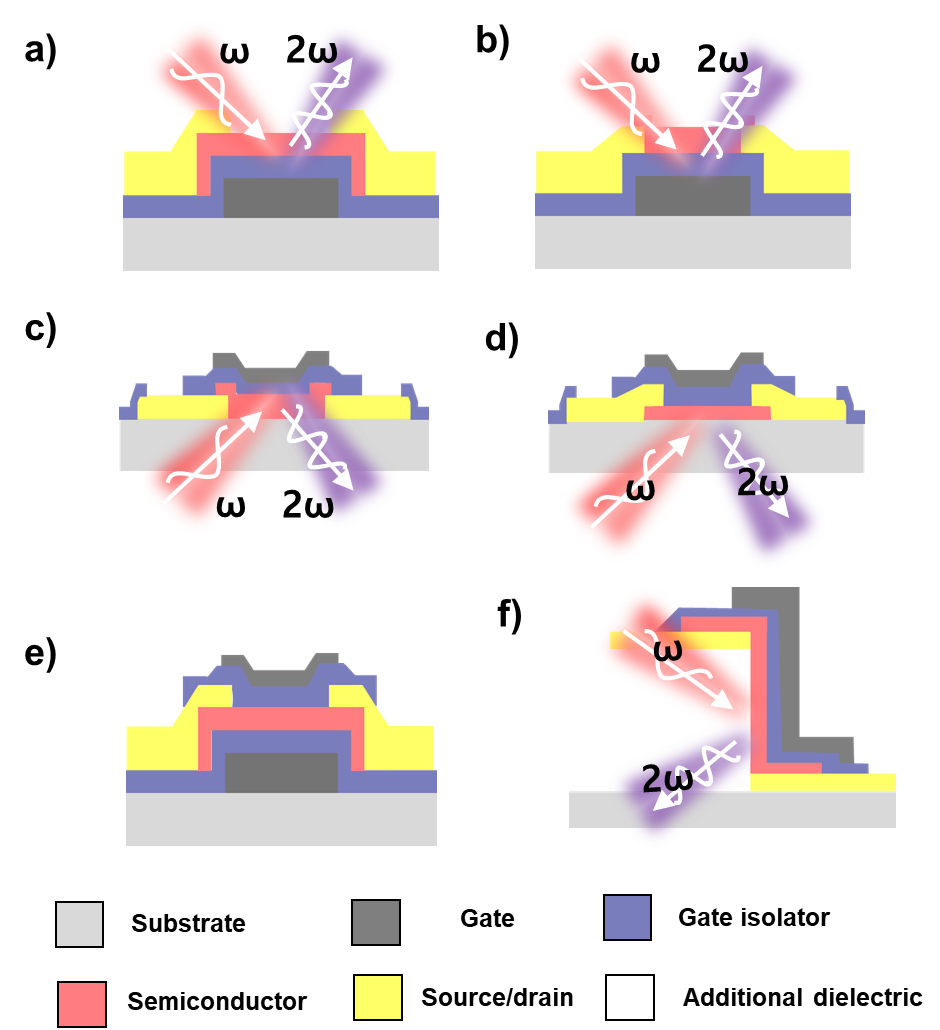


**Supporting Fig. S10.** Optical access for SHG intensity measurement in various TFT structures for display panels: a) Bottom-Gate, Top-Contact (BG-TC); b) Bottom-Gate, Bottom-Contact (BG-BC); c) Top-Gate, Bottom-Contact (TG-BC); d) Top-Gate, Top-Contact (TG-TC); e) Double-Gate; f) Vertical TFT structure.

**Supporting material 11.**

**
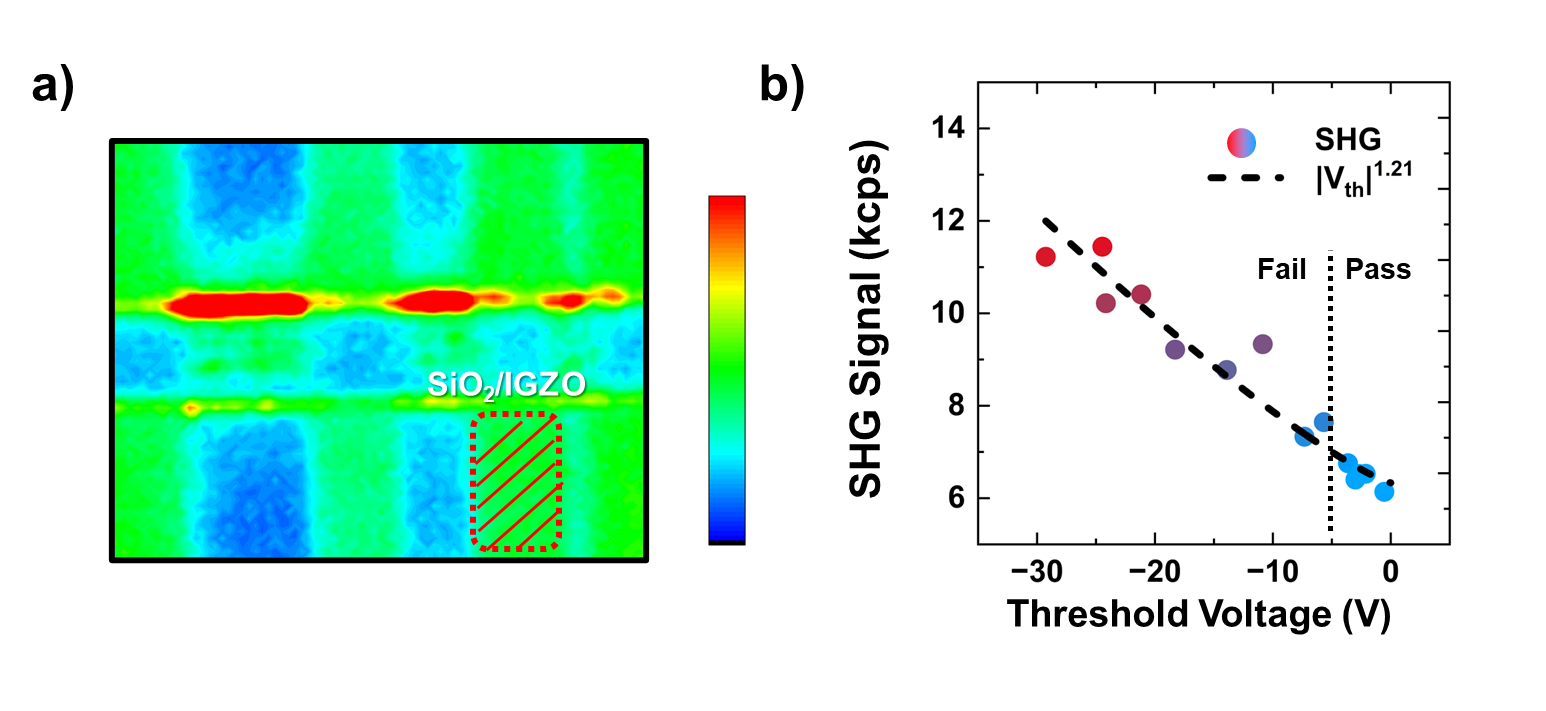
**

**Supporting Fig. S11.** a) SHG image of devices. Both the Al/SiO_2_/IGZO and SiO_2_/IGZO areas emit SHG signals. b) The relationship between V_TH_ and the SHG signal. The SHG intensity from SiO_2_/IGZO shows a similar relationship to V_TH_.

To demonstrate the feasibility of measuring electrical properties through SHG intensity in AOS/GI structures, we investigate the correlation between device performance and SHG intensity from AOS/GI areas. Although there are differences in background signals and scaling factors compared to the results in Figure 6 a), a similar relationship is observed between the SHG signal from the AOS/GI area and V_TH_. Therefore, it is possible to measure electrical properties after GI deposition in top-gate structures.
